# Defining lipids and T cell receptors involved in the intrinsic allergenicity of nut proteins

**DOI:** 10.1186/s13601-020-00358-3

**Published:** 2020-11-25

**Authors:** Rui Wang, Ashfaq Ghumra, Stella Cochrane, Lucy Fairclough, Richard Broughton, Louise V. Michaelson, Frederic Beaudoin, Marcos J. C. Alcocer

**Affiliations:** 1 School of Biosciences, University of Nottingham, Sutton Bonington Campus, Loughborough, LE12 5RD UK; 2 School of Life Sciences, University of Nottingham, Nottingham, NG7 2RD UK; 3 Unilever Safety and Environmental Assurance Centre (SEAC), Colworth Science Park, MK44 1LQ Sharnbrook, UK; 4 Plant Sciences Department, Rothamstead Research, Harpenden, AL5 2JQ UK

**To the Editor**

What makes a protein allergenic, and in particular a food allergen, has not yet been defined. Previously, using model systems, it has been shown plant lipids have an essential role in the allergenicity of the Brazil nut allergen Ber e 1 and NKT-like cells are involved in the sensitisation phase to nut proteins [1–3]. Further progressing investigation of these findings we hereby share details of work to improve and optimise protocols for isolation of lipid responsive human NKT-like cells, sequence and express lipid-binding T cell receptors (TCRs) and use these TCRs to screen Brazil nut lipid fractions.

Primary NKT cells (CD3+, CD56+) from 4 allergic and 2 healthy human volunteers, were targeted by FACS. The NKT cells were challenged with lipids, active cells individually sorted and α/β and γ/δ TCR sequences amplified. The lipid-activated specific populations of TCRs were then identified, sequenced and cloned into expression constructs for use in the in-vitro system shown in Fig. 1. The CD3 + CD56 + lymphocytes were co-cultured with 5 µg/ml controls (without lipid or α-GalCer ) or 1 × 10^6^ lipid-mixture loaded APC cells (MUTZ3) as previously described [4]. Single CD3 + CD56 + CD69 + high cells were isolated by FACS and TCR pairs sequenced and characterised as described [5, 6]. From this screening around 103 pairs of TCR DNA sequences were obtained that were analyzed and classified against human TCR sequence libraries using the IMGT/V-QUEST website.

**Fig. 1:**
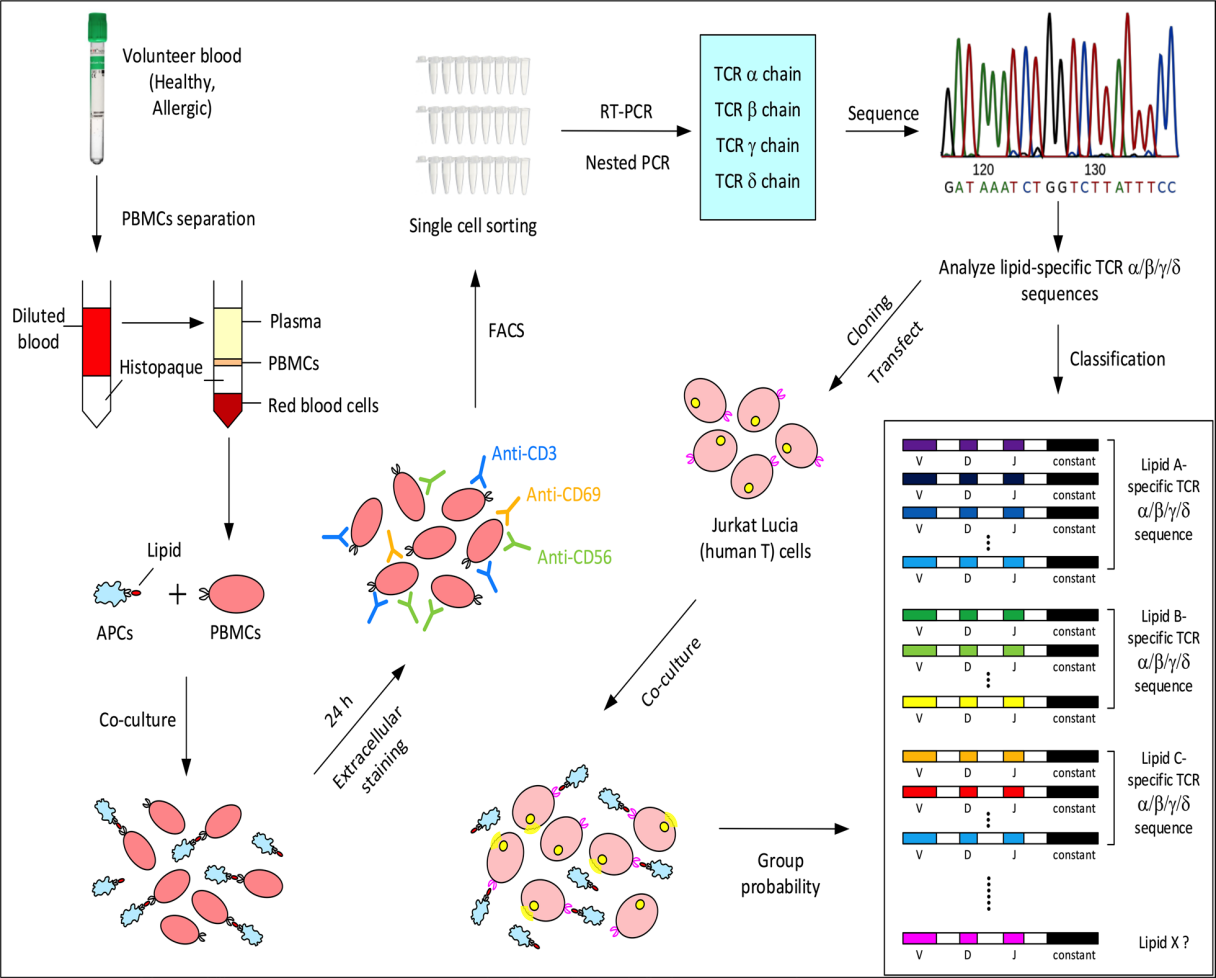
Procedure overview. Overview of the multiplex PCR protocol employed for the separation and characterisation of CD3 + CD56 + CD69 + high NKT-like lymphocytes responsive to lipids. PBMC from volunteers (healthy-Medical School Ethics BS25062015 and allergic Nottingham Health Science Biobank (Ethics: ACP223)) were co-cultured with lipid loaded MUTZ3. TCRs from single CD3 + CD56 + CD69 + high cells were FACs isolated, sequenced [5, 6], and cloned into expression plasmids [4]. Jurkat 76 cells were then transfected for functionality tests as previously described [4]

From the 103 pairs of sequences, some containing the Jα-33 marker for MAIT cells, three TCR pair sequences (1 α/β and 2 γ/δ) were identified within the nut group that were not present in the controls. These 3 TCR pairs were cloned into acceptor bidirectional plasmids as previously described [4], named as pMJA290, pMJA295 and pMJA297 and filed into Genbank as MK764035, MK764036, MK764037. In an attempt to detect the functions of these 3 TCRs, a plasmid (pMJA219) containing the α-GalCer responsive human TRAV10 and TRBV25 sequences was constructed. The α/β TCR sequences used in pMJA219 (TRAV10/TRBV25) design have been previously described as α-GalCer-specific using lipid loaded tetramers [4]. pMJA219 (TRAV10/TRBV25) containing a synthetic TCR sequence specific to α-GalCer was used as control.

To investigate which lipid(s) were recognized by the TCR sequences from allergic patients, nut lipids were fractionated in three major classes: neutrolipids (NL), glycolipids (GL) and polar lipids (PL) as described [7]. These lipid classes were further fractionated by thin layer chromatography (TLC). 9 TLC fractions were isolated from the PL class (Fig. 2a) and preparative solutions individually loaded onto MUTZ3 cells for presentation in co-culture to T cells transiently transfected with pMJA290, pMJA295 and pMJA297 plasmids. As shown in Fig. 2b, in transient Jurkat 76 transfection experiments [4] expressed TCRs from pMJA295 and 297 loaded with lipids from fraction PL5, PL6, PL7 and PL8 from Brazil nut preferentially activate the surrogate T cell with release of IL-2. These two TCR plasmids induced increased IL-2 expression in response to nut lipids but not α-GalCer which displayed a response similar to the no TCR and no lipid treatment controls (Fig. 2b: J76/no lipid). In these lipid screening experiments 2 × 10^5^ surrogate T cells were transfected with 1 µg plasmid carrying one of the TCR sequences and co-cultured with 2.5 × 10^4^ APCs loaded with 5 µg/ml Brazil nut polar lipids fractions. Data were analysed using Microsoft Excel.

**Fig. 2:**
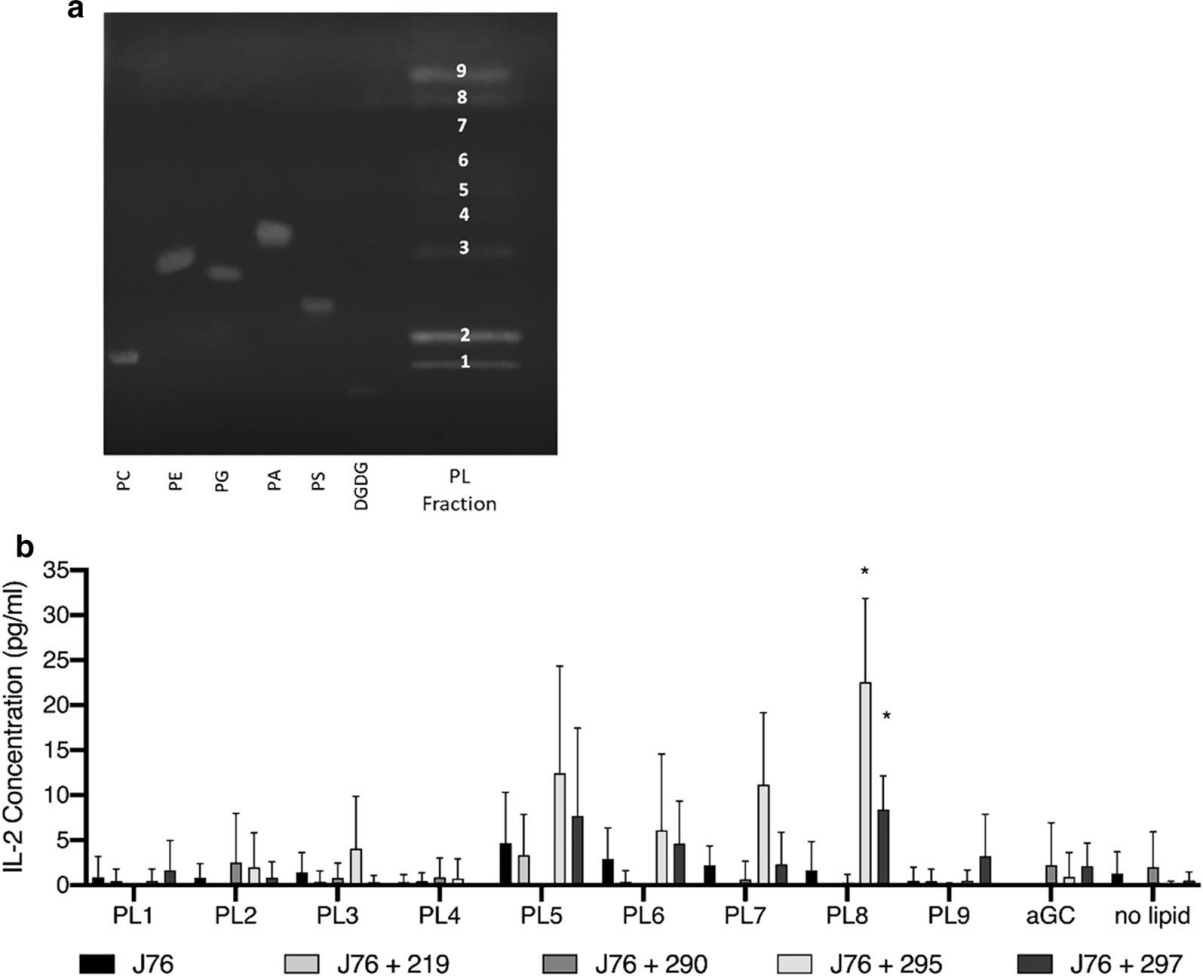
Fractionation of Brazil nut polar lipid (PL) by TLC (**a**) and in vitro TCR specific activation (**b**). IL-2 release of co-culture containing MUTZ3 and pMJA219 (TRAV10/TRBV25), pMJA290, 295 and 297 Jurkat 76 (J76) transient sequences experiments using nut lipid fractionated polar lipids (PL). Molecular standards on TLC: PC: Phosphatidylcholine; PE: Phosphatidylethanolamine; PG: Phosphatidylglycerol; PA: Phosphatidic acid; PS: Phosphatidylserine; DGDG: Digalactosyl Diacylglycerol. The identity of PL1, PL2 and PL3 as PC, PI and PE respectively was confirmed by ESI-MS/MS. All experiments were set up in triplicate wells and repeated three times. Error bars indicate standard deviation. P values indicate significance level as assessed by T test (*p < 0.05)

Brazil nuts contain over 70% (wt/wt) oil mainly composed of triacylglycerol molecules (TAG) with phospholipids (PLs) representing less than 1% of total nut lipids. Furthermore, fractions PL5-8 are minor components of Brazil nut PLs which are dominated by 4 lipids visualized as bands PL1-4 on TLC (Fig. 2a). The composition of these fractions was investigated by Electrospray Ionisation-tandem Mass Spectrometry (ESI-MS/MS) using an accurate mass Orbitrap instrument and molecular structure database.

pMJA295 and 297 TCRs displayed stronger activation with PLs in fractions PL5-PL8 but only the response to PL8 was statistically significant. PL8 is enriched in a single molecular species detected both as a free form (m/z 714.5563, C41H79O7P) and as a lithium adduct (m/z 721.5718, C41H79O7PLi). The identity of this compound was investigated using two molecular structure database search tools: http://www.lipidmaps.org/resources/tools/bulk_structure_searches.php?database=LMSD and http://alex123.info/ALEX123/MS.php. Two compounds were consistently suggested depending on the positive neutral or negative detection mode: glucosylceramides HexCer(34:2) (C40H75NO9) in positive [M + H] + and HexCer(t34:1) (C40H77NO9) in negative [M-H]- modes or an ether phosphatidic acid compound PA(O-38:2) (C41H79O7P) in neutral mode. Comparison with molecular markers suggests the position of PL8 on TLC is more compatible with an ether lipid molecule than glucosylceramides.

These results suggest specific classes of nut lipids might be involved in activation (CD69+) of CD3 + CD56 + cells from nut-allergic patients and that α/β and γ/δ TCRs sequences such as pMJA297 and 295 might be involved in the lipid recognition. These results complement previous findings that Ber e 1 can accommodate one lipid molecule (stoichiometry 1:1; K_d_ of 5.6 ± 0.1 µM) as demonstrated in ANS titration and NMR exchange experiments [1, 8]. However, whether the lipids described here are bound to Ber e 1 remains to be demonstrated.

Although exciting, these results are preliminary and do not unequivocally demonstrate the nut lipid identified is the differentiating factor between a protein (Ber e 1) able to sensitise and an inactive one. However, taken together these results further support our initial hypothesis that natural plant lipid might play an essential role in the intrinsic allergenicity of the nut major allergen Ber e 1.

The putative active lipids described here, the campothecin cross-linked lipid described for Pru p 3 [9] and the collective lipid ligands described in many other systems and reviewed elsewhere [10] are now essential components of the discussion on intrinsic allergenicity of proteins.

In conclusion, we improved and optimised protocols for the isolation of lipid responsive human NKT-like cells, sequenced and expressed lipid binding TCRs and used these TCRs to screen Brazil nut lipid fractions. The results from this study help to characterise the intrinsic factors linked to Ber e 1 allergenicity and to define what makes a common protein within a food matrix context, allergenic to a particular group of susceptible individuals.

## Data Availability

The datasets used and/or analysed during the current study are available from Dr Rui Wang (ruiwang316@outlook.com) on reasonable request.
